# The relevance of the Siewert classification in the era of multimodal therapy for adenocarcinoma of the gastro-oesophageal junction

**DOI:** 10.1002/jso.23484

**Published:** 2013-11-14

**Authors:** Nathan J Curtis, Fergus Noble, Ian S Bailey, Jamie J Kelly, James P Byrne, Timothy J Underwood

**Affiliations:** 1 Department of Surgery, University Hospital Southampton NHS Foundation TrustSouthampton, Hampshire, UK; 2 Cancer Sciences Unit, Faculty of Medicine, University of Southampton, University Hospital Southampton NHS Foundation TrustSouthampton, UK

**Keywords:** Siewert classification, oesophageal cancer, oesophagectomy, surgical approach

## Abstract

**Introduction:**

The Siewert classification has been used to plan treatment for tumours of the gastro-oesophageal junction since its proposal in the 1980s. The purpose of this study was to assess its continued relevance by evaluating whether there were differences in the biology and clinical characteristics of adenocarcinomas by Siewert type, in a contemporary cohort of patients, in whom the majority had received neoadjuvant chemotherapy.

**Methods:**

A prospective database was reviewed for all patients who underwent resection from 2005 to 2011 and analysed with regard to Siewert classification determined from the pathological specimen, treatment and clincopathological outcomes.

**Results:**

Two hundred and sixteen patients underwent oesophagogastric resection: 133 for type I, 51 for type II and 33 for type III tumours. 135 Patients (62.5%) received neoadjuvant chemotherapy with no difference between groups. There were no significant differences in age, sex, pT stage, pN stage, pM stage, ASA, or inpatient complications between patients with adenocarcinoma based on their Siewert classification. There was a significant increase in maximum tumour diameter (*P* = 0.023), perineural invasion (*P* = 0.021) and vascular invasion (*P* = 0.020), associated with more distal tumours (Type III > Type II > Type I). Median overall survival was significantly shorter for more distal tumours (Type I: 4.96 years vs. Type II: 3.3 years vs. Type III: 2.64 years; *P* = 0.04). The surgical approach did not influence survival.

**Conclusion:**

In the era of multi-modal treatment pathological Siewert tumour type is of prognostic value, as patients with Type III disease are likely to have larger and more aggressive tumours that lead to worse outcomes. *J. Surg. Oncol*. 2014;109:202–207.

## INTRODUCTION

Since the 1980s, the Siewert classification has been used to plan treatment for adenocarcinomas arising from the gastro-oesophageal junction (GOJ). In an attempt to promote diagnostic homogeneity, Siewert described a system based on the relationship between the tumour origin and the GOJ evaluated at endoscopy prior to resection 1. Tumours whose epicentre was in the distal oesophagus were grouped as Siewert I, carcinoma immediately arising at the GOJ were considered Siewert II and subcardial carcinoma of the fundus called Siewert III. This classification has subsequently been used in staging and selection of the surgical approach to tumour resection 2. Many aspects of attempting GOJ tumour classification have attracted criticism. The GOJ is an artificial division between two organs that remains difficult to accurately localise at endoscopy, radiologically or by laparoscopic assessment and inter-observer divergence has been shown 3–5. The presence of Barrett's oesophagus, hiatus hernia or the tumour itself may distort the anatomical findings. Also, large tumours may straddle two Siewert groups and the epicentre may be hard to define 3. Patterns of lymph node spread have been shown by some to be similar for GOJ and distal oesophageal tumours 3,6. However, when major treatment decisions are based on Siewert group, such as surgical approach 7, the risk of incomplete resection through inadequate lymphadenectomy exists if the tumour is incorrectly classified 3. Some groups advocate a transthoracic two-field resection for GOJ adenocarcinoma irrespective of Siewert group and have demonstrated similar tumour biology and patient survival between tumours of the distal oesophagus and GOJ 3. Others would advocate a tailored approach to GOJ tumours with the belief that Siewert III tumours represent true gastric cancer and are better treated with total gastrectomy and D2 lymphadenectomy 8.

The most recent revision of the Tumour, Node, Metastasis Classification system (TNM7), for oesophageal cancer has attempted to bring uniformity to the assessment of GOJ tumours. TNM7 9 classifies all tumours within 5 cm of the GOJ which extend into the oesophagus as oesophageal and makes no attempt to subclassify tumours based on their anatomical topographical origin 10. TNM7 was developed using complex computational modelling in an attempt to provide accurate prognostication for each homogeneous stage group 11. However, the dataset included mostly patients treated with surgery alone and was based on pathological staging.

The multidisciplinary management of GOJ tumours has evolved since the introduction of the Siewert classification. Whilst widespread improvements in pre-operative staging 12, patient selection 13, critical care 14, nutritional support and surgical techniques 15 including laparoscopic surgery 16 have been made, arguably the largest single change in the management of GOJ tumours has been the application of neoadjuvant therapy 17. Multimodal treatment has been reported to increase R0 resection rates through tumour down staging 18,19, decrease the number of involved lymph nodes 20,21 and improve long-term survival compared to surgery alone 17–19,22–24. Pathological complete response (pCR) to chemoradiotherapy has been reported 25,26 with selected studies demonstrating pCR in around 20–30% of patients 18,21. Neoadjuvant treatments for GOJ tumours are now in widespread use around the world.

The changes made in TNM7 and the widespread use of neoadjuvant therapies for GOJ tumours questions the relevance of a classification that was established when most tumours were treated with surgery alone. Therefore, the purpose of this study was to evaluate whether there were differences in the biology and clinical characteristics of adenocarcinomas of the GOJ when classified by Siewert type, in a contemporary cohort of patients receiving multi-modal therapy.

## METHODS

A prospectively collected database of consecutive patients undergoing oesophagogastric resection for tumours of the GOJ treated at a single UK university teaching hospital between January 2005 and December 2011 was reviewed. All patients were discussed at a specialist multidisciplinary team meeting. Standard staging investigations included endoscopic ultrasonography, high-resolution computed tomography, integrated fluorodeoxyglucose positron emission tomography/computed tomography (PET-CT) and staging laparoscopy where indicated. Patients considered suitable for surgical resection with tumours staged as T2 N0 M0 or above were considered for neoadjuvant chemotherapy that was uniformly applied irrespective of tumour location. Our regime consists of three 21-day cycles of ECF (Epirubicin 50 mg/m^2^, Cisplatin 60 mg/m^2^, both intravenously on day 1 and protracted venous infusion 5-FU 200 mg/m^2^ per day) or ECX (Epirubicin 50 mg/m^2^, Cisplatin 60 mg/m^2^, both intravenously on day 1 and Capecitabine 625 mg/m^2^ orally twice daily for 21 days) or EOX (Epirubicin 50 mg/m^2^ i.v. bolus and Oxaliplatin 130 mg/m^2^ i.v. infusion over 2 hr on day 1, Capecitabine 625 mg/m^2^ orally twice daily for 21 days).

In our unit, based on pre-operative assessment, Siewert type I and II tumours are treated as oesophageal cancer, with transthoracic procedures. Type III tumours are treated as gastric cancers with an abdominal approach, typically, total gastrectomy, distal oesophagectomy and D2-lymphadenectomy. All patients considered to have a type III tumour pre-operatively underwent staging laparoscopy. Types of oesophagogastrectomies included Ivor-Lewis, left thoracoabdominal with or without cervical anastomosis, and transhiatal oesophagogastrectomy or minimally invasive oesophagogastrectomy either 2 stage (MIO-2) or 3 stage (MIO-3). Patients were cared for by a specialist oesophagogastric team who applied a similar perioperative regime to all.

Patients were routinely followed-up for 5 years post-surgery and were also seen on as required basis if symptomatic. Recurrence of disease during follow-up was defined as the first site or sites of recurrence with radiological or pathological confirmation. Site of recurrence was defined as local: anastomosis or local lymph nodes, nodal: regional lymph nodes and distant: distant nodal or distant organ recurrence.

Data recorded included demographics, tumour characteristics, type of resection, histopathological analysis of the surgical specimen, post-operative complications and mortality. Classification systems used for analysis included TNM7 8,9, Clavien-Dindo 27, tumour regression grade (TRG) 28 and Siewert 1 using the final tumour site determined from the pathological specimen. Pathological tumour clearance (‘R’-status) was determined according to the Royal College of Pathologists of England system.

Kruksal–Wallis, Mann–Whitney *U* and Pearson's *χ*^2^ tests were used. A *P*-value < 0.05 was considered significant. Overall survival was analysed by the Kaplan–Meier method calculated from the date of operation until the date of death excluding inpatient deaths (n = 4) and R1 resections (n = 42). Statistical analysis was performed with SPSS® version 19 (SPSS, Chicago, IL).

## RESULTS

Two hundred and sixteen patients underwent oesophagogastric resection: 132 for type I, 51 for type II and 33 for type III tumours. One hundred and thirty-five (62.5%) underwent neoadjuvant chemotherapy prior to resection. The majority of patients were male (85.6%) but there were no significant differences in age, sex, ASA or neoadjuvant use between patients with adenocarcinoma based on their Siewert classification (Table1). Barrett's oesophagus was observed most commonly in association with type I tumours (Type I 58.3%, Type II 21.6%, Type III 9.1%; *P* < 0.0001).

**Table I:** Patient Demographics, *American Society of Anesthesiologists Physical Status Classification System* Score and Presence of Barrett's Metaplasia, Treatment Plans, Surgical Approach and Operation Performed by Siewert Groups

	Siewert I (n = 132)					Siewert II (n = 51)					Siewert III (n = 33)					*P*-value
	Median	Min	Max	Count	Column, N %	Median	Min	Max	Count	Column, N %	Median	Minimum	Maximum	Count	Column, N %	
Age at Op	67.6	42.1	84.1			64.3	32.8	82.5			67.6	50.7	85.4			ns
Sex																
Male					113	85.6%			45	88.2%				27	81.8%	ns
Female					19	14.4%			6	11.8%				6	18.2%	
Barrett's																
Yes					77	58.3%			11	21.6%				3	9.1%	
No					55	41.7%			40	78.4%				30	90.1%	
ASA																
1					8	6.1%			3	5.9%				3	9.1%	ns
2					100	76.3%			37	72.5%				23	69.7%	
3					23	17.6%			11	21.6%				7	21.2%	
Treatment																
Surgery only					51	38.6%			18	35.3%				12	36.4%	
Neoadjuvant chemotherapy and surgery					81	61.4%			33	64.7%				21	36.6%	
Transthoracic vs. abdominal																
Transthoracic					127	96.2%			50	98.0%				15	45.5%	<0.0001
Abdomen					5	3.8%			1	2.0%				18	54.5%	
Operation type																
Ivor-Lewis					33	25.0%			20	39.2%				3	9.1%	<0.0001
Minimally invasive oesphagectomy—2 stage					46	34.8%			17	33.3%				7	21.2%	
Minimally invasive oesphagectomy—3 stage					20	15.2%			5	9.8%				3	9.1%	
Left thoracoabdominal					28	21.2%			8	15.7%				2	6.1%	
Laparoscopic gastrectomy + distal oesophagectomy					0	0.0%			1	2.0%				4	12.1%	
Open D2 gastrectomy + distal oesophagectomy					5	3.8%			0	0.0%				14	42.4%	

Surgical approaches varied by Siewert group (Table1). As determined by analysis of the pathological specimen, 96.2% of Siewert I tumours had a transthoracic procedure compared to 45.5% for type III tumours. Of the 33 Siewert III specimens, 14 (42%) were pre-operatively staged as more proximal disease and all of these patients underwent a transthoracic operation. Seventy-eight percent of Type III tumours resected via an abdominal approach were staged as T3 or T4 on the pathological specimen, compared with 33% via a transthoracic approach (*P* = 0.04). However, there were no other significant differences observed for Type III tumours dependent on surgical approach (p or ypN-stage, nodal yield, R0/R1 resection rate, anastomotic leak rate, post-operative complications or survival). Local, nodal and distant recurrences were more common in distal tumours (Table2). One hundred and forty-one (65.2%) cases were performed laparoscopically. The surgical approach did not impact on the frequency or severity of post-operative complications. Patients (22.6%) developed a major complication (CD 3–5). The overall anastomotic leak rate was 7.4%, with no differences between surgical approach or Siewert tumour type. Four inpatient deaths (1.85%) were recorded, all following transthoracic surgery (Table3).

**Table II:** Tumour Recurrences by Location

	Tumour site by Siewert classification					
Siewert I (n = 132)		Siewert II (n = 51)		Siewert III (n = 33)	
Local recurrence						
Yes	6	4.5%	1	2.0%	2	6.1%
No	126	95.5%	50	98.0%	31	93.9%
Nodal recurrence						
Yes	10	7.6%	2	3.9%	6	18.2%
No	122	92.4%	49	96.1%	27	81.8%
Distant recurrence						
Yes	34	25.6%	18	35.3%	8	24.2%
No	98	74.4%	33	64.7%	25	75.8%

*P* = ns.

**Table III:** Post-Operative Complications and Anastomotic Leak Data

	Transthoracic vs. abdominal				*P*-value
Transthoracic		Abdomen			
Major or minor					
Minor or no comp	145	76.0%	21	87.5%	ns
Major or death	46	24.0%	3	12.5%	
Clavien Dindo Classification					
No complication	69	35.9%	11	45.8%	ns
Grade 1	12	6.3%	1	4.2%	
Grade 2	64	33.3%	9	37.5%	
Grade 3	22	11.5%	1	4.2%	
Grade 4	21	10.9%	2	8.3%	
Grade 5	4	2.1%	0	0.0%	
None or minor or major complication					
No	69	35.9%	11	45.8%	ns
Minor	77	40.1%	10	41.2%	
Major	46	24.0%	3	12.5%	

	Siewert I		Siewert II		Siewert III		*P*-value
Anastomotic leaks							
Yes	10	7.6%	4	7.8%	2	6.3%	ns
No	122	92.4%	47	92.2%	31	93.9%	

Histopathological assessment showed no differences in tumour differentiation, p or ypT (*P* = 0.080), p or ypN (*P* = 0.367), number of positive lymph nodes, p or ypM (0.828) or R1 resections between the groups (Table4). 19.4% of Resections were classified as R1 using the Royal College of Pathologists of England system. More distal tumours were significantly bigger (mean tumour diameter, Type I: 25.8 mm, Type II: 33.1 mm, Type III: 35.6 mm, *P* = 0.023), more likely to show vascular (*P* = 0.02) and perineural invasion (*P* = 0.021) and were associated with a higher lymph node harvest (median nodal harvest; Type I: 17, Type II: 20, Type III: 23; *P* = 0.004), although the number of lymph node metastasis did not differ between tumour types. In 25%, of patients treated with neoadjuvant chemotherapy significant tumour response was observed in the resected specimen (TRG 1–2) and there was no difference in the likelihood of observing tumour regression based on Siewert tumour type (*P* = 0.676).

**Table IV:** Histopathological Analysis Results

		Siewert I (n = 132)					Siewert II (n = 51)					Siewert III (n = 33)					*P*-value
		Median	Min	Max	Count	N %	Median	Min	Max	Count	N %	Median	Minimum	Maximum	Count	N %	
pT TNM7	T0				6	4.5%				1	2.0%				2	6.1%	ns (0.08)
	T_in situ/high grade dysplasia_				35	26.5%				7	13.7%				4	12.1%	
	T1				28	21.2%				15	29.4%				8	24.2%	
	T2				61	46.2%				26	51.0%				13	39.4%	
	T3				1	0.8%				2	3.9%				6	18.2%	
	T4				1	0.8%				0	0.0%				0	0.0%	
pN TNM7	N0				74	56.1%				23	45.1%				15	45.5%	ns
	N1				23	17.4%				12	23.5%				5	15.2%	
	N2				17	12.9%				12	23.5%				7	21.2%	
	N3				18	13.6%				4	7.8%				6	18.2%	
pM TNM7	M0				129	97.7%				49	96.1%				32	97.0%	ns
	M1				3	2.3%				2	3.9%				1	3.0%	
Nodes +ve		0	0	20			1	0	15			1	0	24			ns
Nodal yield		17	3	52			20	7	53			23	7	49			0.004
Pathological tumour clearance	R1				27	20.5%				11	21.6%				4	12.1%	ns
	R0				105	79.5%				40	78.4%				29	87.9%	
Vascular invasion	Yes				30	22.7%				20	39.2%				14	42.4%	0.020
	No				102	77.3%				31	60.8%				19	57.6%	
Lymphatic invasion	Yes				17	12.9%				9	17.6%				4	12.1%	
	No				115	87.1%				42	82.4%				29	87.9%	
Perineural invasion	Yes				10	7.6%				11	21.6%				6	18.2%	0.021
	No				122	92.4%				40	78.4%				27	81.8%	
Differentiation/grade	Grade 1				16	12.1%				4	7.8%				1	3.0%	ns
	Grade 2				39	29.5%				20	39.2%				8	24.2%	
	Grade 3				77	58.3%				26	51.0%				24	72.7%	
	Grade 4				0	0.0%				1	2.0%				0	0.0%	

**Fig 1:**
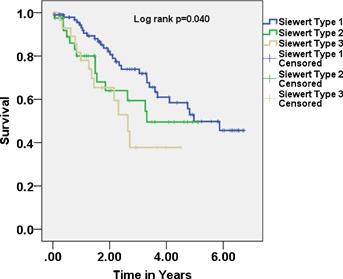
Kaplan–Meier graph showing overall survival by Siewert grouping (*P* = 0.04).

Median follow-up was 2.94 years. Median overall survival for the full cohort was 3.4 years (95% confidence interval (CI) 2.14–4.66). Median overall survival was significantly shorter for more distal tumours (Type I: 4.96 years (95% CI: 4.12–5.23) vs. Type II: 3.3 years (95% CI: 2.63–4.04) vs. Type III: 2.64 years (2.04–3.63); *P* = 0.04). The surgical approach did not influence survival for all tumour types. Three-year overall survival was significantly better for more proximal tumours and decreased for more distal tumours (Type I: 78%, Type II: 60% HR 1.54 (95% CI: 0.81–2.92), Type III: 37% HR 2.28 (95% CI: 1.17–4.45); *P* = 0.011 (Fig. 1)).

## DISCUSSION

Multidisciplinary experience in the management of GOJ adenocarcinomas has progressed since the Siewert classification was first reported. TNM7 now classifies all tumours within 5 cm of the GOJ, which involve the oesophagus as oesophageal, and makes no distinction between tumours that may be considered to arise from adjacent but different organs (the oesophagus and stomach). The results of multiple randomised trials, published since 2000, strongly support the use of peri-operative therapy for tumours of the GOJ with a consequent marked increase in R0 resection rate and long-term survival with an acceptable short-term side effect profile 18,19,22–24. In our centre, the majority of patients (65.2%) who are suitable for curative resection received multimodal therapy. Given this differs from the 17% in the original Siewert reports 2 our study is relevant for contemporary practice.

In contrast to previous reports, Type I tumours made up 61% of our cohort. Siewert and Leers have previously reported 37% 2 and 50% 3 respectively from large single centre western series compared to the low prevalence of type III tumours seen in eastern series (5.6%) 29. The proportional increase in Type I tumours in our series is likely to represent the widely reported increase in incidence of oesophageal adenocarcinoma 30,31 associated with gastro-oesophageal reflux disease 32 and Barrett's metaplasia 33 in the United Kingdom 34. The expansion of endoscopic Barrett's surveillance strategies is also likely to have increased disease detection and treatment. Similarly, the decrease in type III cases follows the reduction in incidence of true gastric adenocarcinomas observed in the West 35.

We have demonstrated an overall 5-year survival of >40% with the use of multimodal therapy for tumours of the GOJ. This compares well with the outcomes reported in other large single centre series 2,3. We have further demonstrated that survival is worse for more distal tumours; patients with pathologically defined Type III tumours are less than half as likely to be alive at 3 years when compared to distal oesophageal tumours. We are not the first to report a biological difference between tumours at the GOJ 2,3,5,36. In our series, Type III tumours were larger and they were associated with more frequent evidence of perineural and vascular invasion, although this did not translate into more lymph node metastasis. Whilst this may indicate the type III tumours in this series were of a more advanced stage at presentation, this did not reach statistical significance (pT (*P* = 0.080), pN (0.367), pM (0.828) and AJCC stage grouping (*P* = 0.508)). This suggests a possible difference in the biological behaviour of GOJ tumours based on their anatomical origin. In this series Type III tumours were equally likely to recur in a loco-regional setting as they were at distant sites when compared with Type I and II tumours that recurred at distant sites in ∼80–90% of cases. This finding was not dependent on the operative approach taken to Type III tumours.

One possible explanation for this finding is the anatomical setting of the distal oesophagus when compared with the proximal stomach. A tumour whose epicentre is in the distal oesophagus or at the GOJ may be more likely to give rise to symptoms (dysphagia) at an earlier stage in disease evolution than a proximal gastric cancer that invades into the GOJ as it develops. Furthermore, adjacent organs limit the local spread of oesophageal tumours and operable tumours will be resected en bloc with local lymph nodes and surrounding tissue. It is our practice to routinely take a cuff of hiatal tissue and clear the inferior mediastinum onto pericardium anteriorly and aorta posteriorly. Tumours in the abdomen are not bounded in the same way and may spread into the peritoneal cavity. We routinely perform peritoneal lavage for cytology for Type III tumours as part of our pre-operative work-up, but this strategy has been documented to have limited accuracy 37. Another possible explanation for a higher loco-regional recurrence rate for Type III tumours would be inadequate surgery leading to R1 resections. Our R1 resection rate of 19% is based on the definition of an R1 resection from the Royal College of Pathologists of England, using the American system the proportion of R1 resections falls to 10%, an improvement on the 27% reported by Siewert 2 and comparable to the 7% reported by DeMeester and coworkers 3. There were no differences in R1 resection rates between Siewert tumour types.

Given the fact that tumours within 5 cm of the GOJ are now all classified as oesophageal and treated the same pathologically, our findings add to concerns that Siewert III tumours may be biologically different from tumours of the distal oesophagus and GOJ. Epidemiological data supports the concept that GOJ tumours are oesophageal in origin 35 and therefore our histologically proven Siewert III cases may represent true gastric adenocarcinoma. If so, direct comparison with other GOJ tumours for prognostication may be inaccurate. Even if tumours around the GOJ represent similar biological entities our data suggest that Siewert III tumours tend to be larger at presentation and patients with these tumours are far less likely to be alive 3 years after surgery than patients with more proximal disease, despite multimodal therapy. This is the important information for the patients and their families (3-year survival: Type I: 78%, Type II: 60% HR 1.54 (95% CI: 0.81–2.92), Type III: 37% HR 2.28 (95% CI: 1.17–4.45); *P* = 0.011 (Fig. 1)).

Our data should be regarded with caution because although the total number of resections performed was not inconsiderable and the series benefits from originating at a single centre with defined treatment pathways, the number of Type III tumours was relatively small (n = 33). No differences in pT stage were seen between the Siewert groups (*P* = 0.08) but low Siewert III numbers may have resulted in a type II error. Further, insufficient numbers of Type III tumours prevents full risk stratification analysis by disease stage. The pattern of disease reported in this series represents the current trends of GOJ cancer in the United Kingdom and the findings are relevant for the contemporary treatment of adenocarcinoma of the distal oesophagus and GOJ.

This series also highlights one of the major problems with the Siewert classification, the relative inability of experienced oesophageal physicians to accurately distinguish the epicentre of tumours around the GOJ on pre-operative assessment 5,29. In our cohort, 42% of pathologically proven Type III tumours were designated as more proximal disease during the pre-operative work-up. The majority were defined as Type II tumours and therefore underwent a transthoracic procedure. The outcomes for these patients were similar to those who underwent an abdominal approach and this finding would lend support to the belief that all GOJ tumours may be adequately treated by an oesophagectomy 3. It is possible that the true epicentre of the tumour is better revealed after neoadjuvant treatment and we are therefore better able to accurately identify Siewert type on the resected specimen.

Overall, in the era of multi-modal treatment, in an expert centre, the pre-operative Siewert classification is difficult to assess and corresponds poorly to the resected specimen. The surgical approach to Siewert type III tumours of the GOJ in this series did not appear to change short- and long-term outcome. However, knowing the pathological Siewert group is of considerable prognostic value, as patients with progressively more distal disease were seen to have larger and more aggressive tumours that led to worse outcomes. These findings have implications for research and clinical trials as well as prognosis following resection.
